# Aggressive Angiomyxoma of the Vulva: Which Is the Best Management Strategy? Description of a Case Report and Review of Literature of the Last Ten Years

**DOI:** 10.3390/jcm12051726

**Published:** 2023-02-21

**Authors:** Ferdinando Antonio Gulino, Marianna Gulisano, Carla Ettore, Alexandra Giorlandino, Emanuele Russo, Giuseppe Ettore

**Affiliations:** 1 Department of Obstetrics and Gynaecology, Azienda di Rilievo Nazionale e di Alta Specializzazione (ARNAS) Garibaldi Nesima, 95124 Catania, Italy; 2 Department of Pathological Anatomy, Azienda di Rilievo Nazionale e di Alta Specializzazione (ARNAS) Garibaldi Nesima, 95124 Catania, Italy

**Keywords:** aggressive angiomyxoma, mesenchymal tumour, genital tumour, vulvar lesion

## Abstract

Background: Aggressive angiomyxoma is a rare mesenchymal tumour of the genital tract with a high incidence in women of reproductive age. The aim of our work is to understand which is the best management strategy for this condition, starting from the description of a rare case report up to the performance of a narrative review of the literature. Methods: We report a case of a 46-year-old woman who came to our attention because of the growth of a 10-centimetre pedunculated, non-soft, non-tender mass of the left labium majus. She underwent surgical excision, and the histologic diagnosis was aggressive angiomyxoma. Due to a lack of tumour-free margins, radicalization surgery was carried out after three months. A review of the literature of the last ten years was performed following the PRISMA statement on MEDLINE (PubMed). We obtained data from twenty-five studies describing thirty-three cases. Results: Aggressive angiomyxoma is characterized by a high recurrence rate of between 36 and 72% after surgery. There is no universal consensus about hormonal therapy, and most studies (85%) describe surgical excision followed by only clinical and radiological follow-up. Conclusion: Wide surgical excision is the gold-standard treatment for aggressive angiomyxoma, succeeded by either clinical or radiological (ultrasound or MRI) follow-up.

## 1. Introduction

Aggressive angiomyxoma (AAM) is a locally aggressive mesenchymal tumour arising from the connective tissue of the perineum or the lower pelvis. It predominantly occurs in women of reproductive age, with a female-to-male ratio of 6.6/1 [1].

The main features include a non-soft mass, large multilobular or polypoid or swelling, slow growth, and a slightly circumscribed and gelatinous appearance on the cut section [2].

The pelvis, perineum, vulva, vagina, and bladder are the main anatomic sites involved. Considering its similar clinical presentation to common lesions such as Bartholin duct cysts, lipoma, vulvar mass or vulvar abscess, Gartner duct cysts, vaginal cysts, and vaginal prolapse, this condition is often misdiagnosed [3].

It has a variable size, although usually with a 10–20 cm maximum diameter, and may cause pressure on adjacent organs.

AAM is a slow-growing neoplasm with a high local recurrence rate but limited metastasizing capacity. Cytological investigation shows an absence of nuclear atypia or high mitotic activity. The origin is myofibroblastic differentiation of spindle or stellate cells separated by myxoid stroma and abundant vascular components. The term “aggressive” is used to emphasize the neoplastic character of the blood vessels, its locally infiltrative behaviour, and the high risk of local recurrence [4].

Multiple treatment modalities have been described for the treatment of angiomyxoma. Radical surgery with tumour-free margins is the treatment of choice, but the likelihood of local recurrence is high, despite wide local excision. Adjuvant radiotherapy and chemotherapy are not performed considering the low mitotic activity of angiomyxoma [5].

Radiological investigations are useful for diagnosis and planning of surgery. However, in most cases, the final diagnosis is established by histopathological examination. 

Our study aims to understand which is the best management strategy for this condition, starting from the description of a case of AAM up to the performance of a narrative review of the literature. The description of the case report was performed following the CARE criteria (https://www.care-statement.org/checklist, accessed on 30 December 2022).

## 2. Materials and Methods

### 2.1. Case Report

A 46-year-old Caucasian woman came to the Emergency Obstetric and Gynaecological Department of ARNAS Garibaldi Nesima Hospital in Catania and reported the onset of a pedunculated mass of the left vulva. This pathological condition arose six months before and grew progressively. Her past medical history included three caesarean deliveries, the last of which was 8 years ago, and since then, she had not performed any gynaecological checks. She denied any other pathological condition. First menstruation occurred at the age of 14, followed by a regular rhythm. Her familiar and gynaecologic history was otherwise normal.

**Figure 1:**
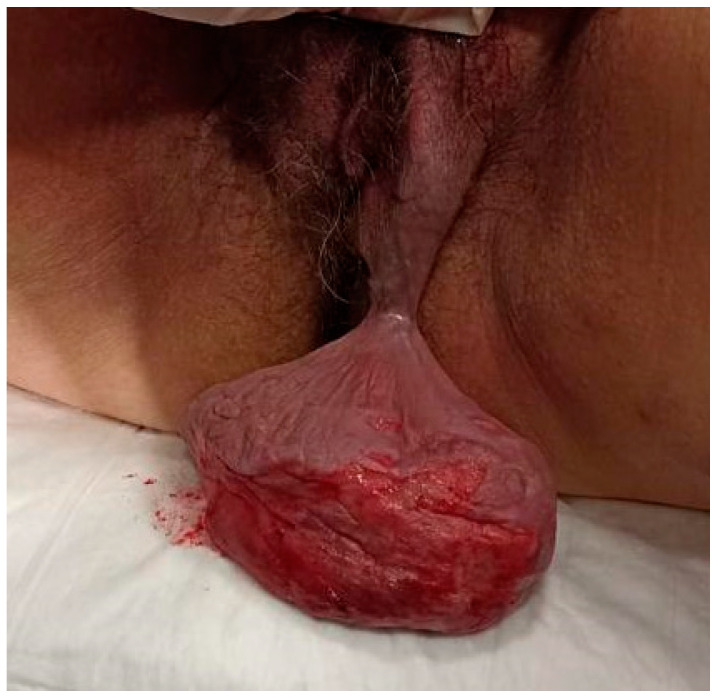
Angiomyxoma of the left labium.

Clinical examination relived a soft, non-tender tumour that was painful upon palpation protruding from the left labium. The size was approximately 10 cm in maximum diameter (Figure 1). The surface appeared irregular upon palpation due to the presence of an area of ulceration of 1.5 cm in the overlying skin in the perineum with a lightly bleeding continuous solution.

The patient complained of regional pain and a feeling of local pressure. There were no detectable inguinal lymph nodes or any urinary symptoms.

Transvaginal ultrasound showed a normal (in size and morphology) uterus and ovaries and the absence of detectable pelvic swellings. Speculum examination was not carried out due to the patient’s discomfort. All laboratory investigations were performed within normal limits.

Surgical transperineal removal of the mass was the immediate approach performed by gynaecologists. The histological piece was removed intact and sent to the Pathology Department for histopathological diagnosis.

**Figure 2:**
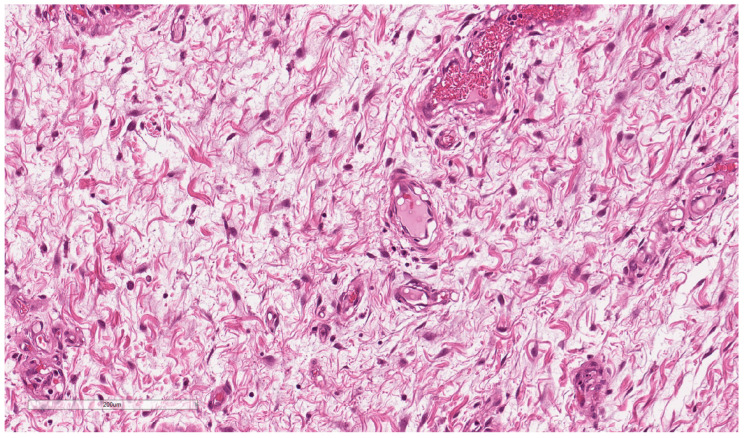
Note the admixture of thin and thick-walled vessels of varying calibre within a sparse cellular stroma.

**Figure 3:**
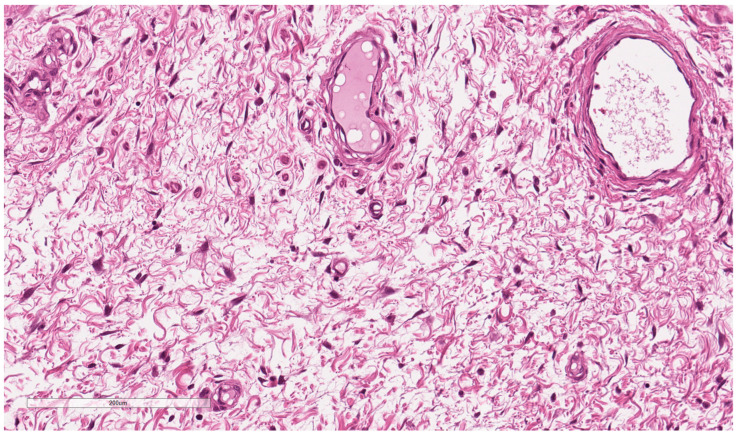
Tumour cells with scanty cytoplasm and bland nuclear features are widely separated by a sparse cellular stroma.

Due to the rarity and complexity of the pathological finding, another pathologist was consulted for a second opinion; ultimately, a diagnosis of aggressive ulcerated angiomyxoma was rendered (Figure 2 and Figure 3). Immunohistochemistry showed tumour cells with 80% ER receptor expression and 5% PR receptor expression.

The analysis revealed positivity for tumour tissue in the surgical resection margins. Pelvic magnetic resonance imaging (MRI) excluded pelvic expansive processes and did not show any persistence (or locoregional recurrence) of the disease.

**Figure 4:**
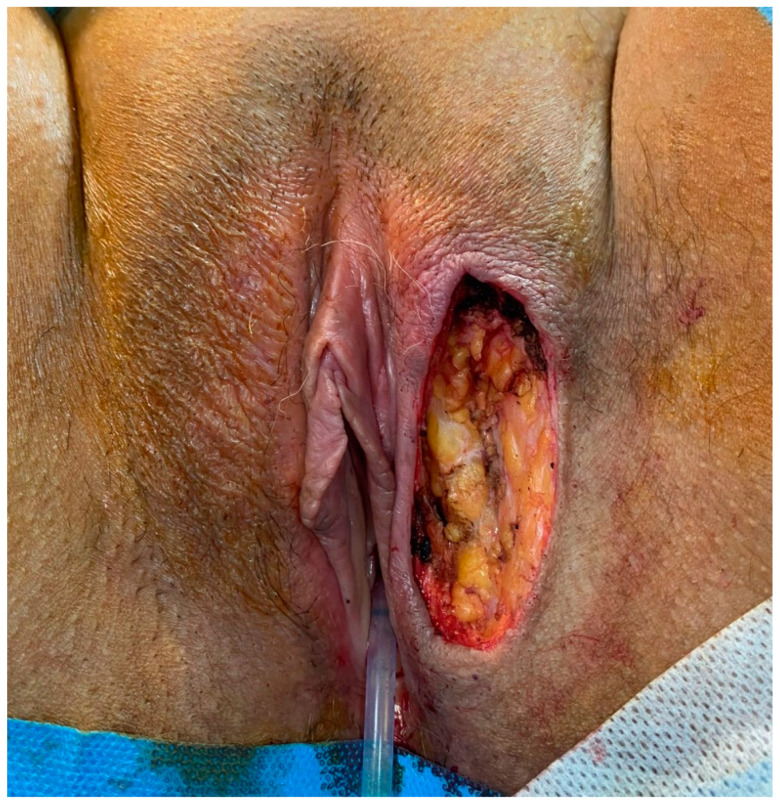
Radicalization three months after primary surgery.

**Figure 5:**
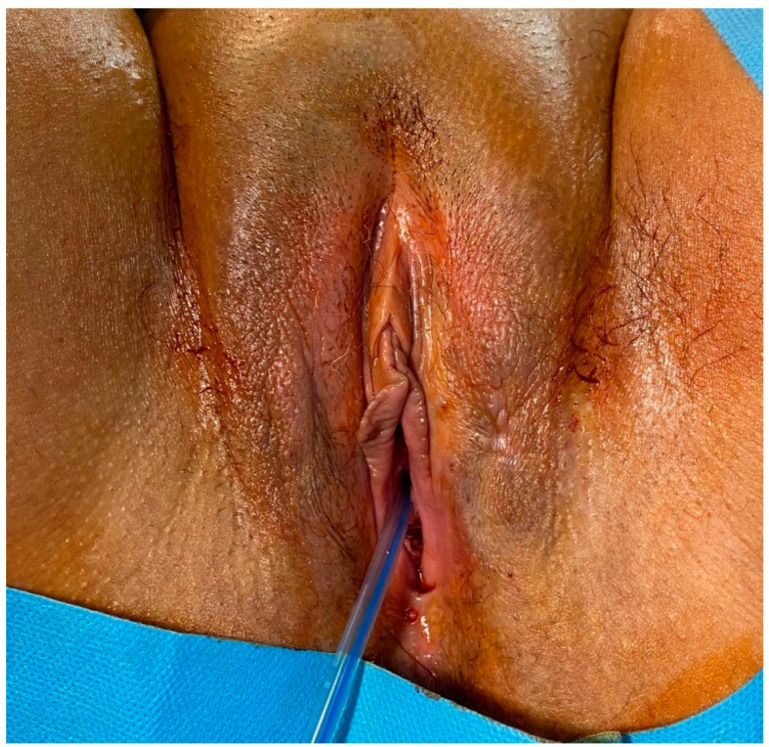
Vulva after radicalization surgery.

In consideration of the lack of tumour-free margins, radicalization surgery was planned three months after primary surgery (Figure 4 and Figure 5). The radicalization was extended up to 20 mm from the previous surgical scar. Clinical follow-up has been regular until now.

### 2.2. Review of Literature

A review of the literature of the last ten years was performed following the PRISMA statement (Preferred Reporting Items for Systematic Reviews and Meta-Analysis) in online MEDLINE (PubMed) database from 2012 to 2022 (Table 1).

**Figure 6:**
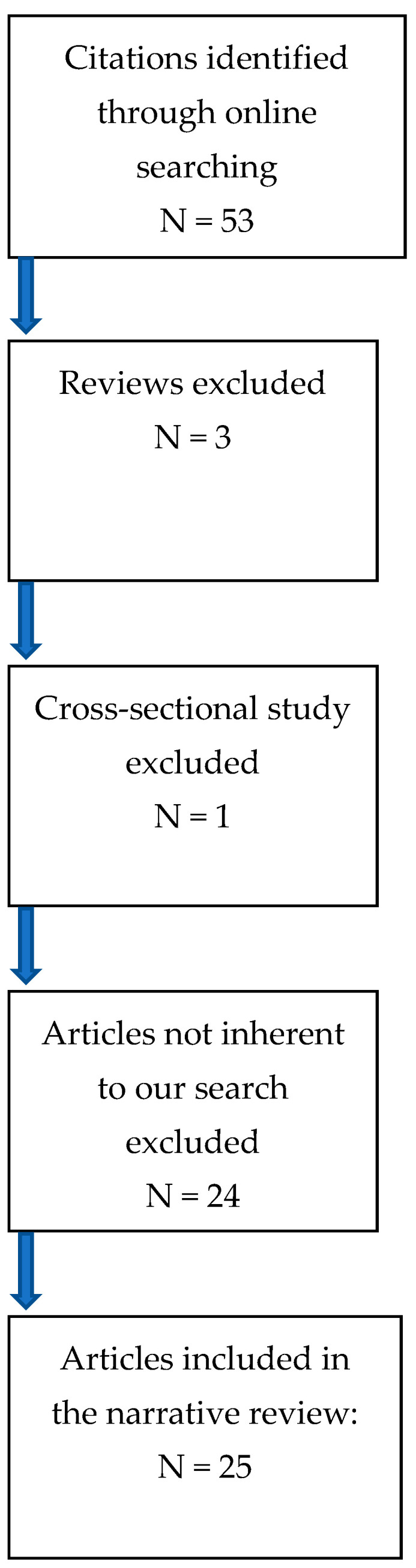
Prisma flow chart.

We used the terms “aggressive angiomyxoma”, “mesenchymal tumour “, “genital tumour”, and “vulva”. Fifty-three citations were identified after online searching. Abstracts/titles were screened by three different authors. Only English-language works were admitted. After initial valuation, we excluded reviews or cross-sectional studies and case reports about items beyond the scope of our eligibility criteria represented by angiomyxoma of the genital tract in women. We also excluded a case of superficial angiomyxoma and studies evaluating data from children, males, or pregnant women due to hormonal interference for tumour growth during pregnancy (Figure 6).

## 3. Results

We conducted a review of case reports of the last 10 years on aggressive vulvar angiomyxoma in non-pregnant women. Twenty-five studies were selected regarding thirty-three cases of aggressive angiomyxoma [6,7,8,9,10,11,12,13,14,15,16,17,18,19,20,21,22,23,24,25,26,27,28,29,30].

The median age of the patients was 39. One case of an 89-year-old woman, despite the patient’s age, was not typical. 

Almost all women complained of the onset of a growing soft, tender mass on the pelvic region without pain or other urinary symptoms (97%). No inguinal lymph nodes were detectable in any of the cases. In just one report, there was an absence of a palpable mass, and diagnosis was achieved only by ultrasound detection of a detached echogenic mass of the pelvic cavity (3%). The principal symptom was a feeling of pelvic fullness (100%). Two patients complained of dysuria (6%), one declared an excess of pruritus (3%), one reported dyspareunia (3%), one reported difficulty urinating (3%), and one noted blood loss from ulceration (3%).

Primary angiomyxoma was described in twenty-five women (75%); the other eight patients were affected by recurrent lesions after a period between 1 and 20 years after primary excision (24%).

The treatment choice in all cases was surgery with wide margins. After excision, five women received hormonal therapy for three or more months represented by GnRH-analogue (15%); others followed only clinical and radiological periodic controls (85%).

## 4. Discussion

Aggressive angiomyxoma (AAM) is a rare mesenchymal tumour described for the first time in 1983 by Steeper and Rosai as a slow-growing neoplasm with a high local recurrence rate but benign behaviour with poor ability to give distant metastasis [4].

It was classified as ‘‘Tumours of uncertain differentiation’’ in the latest WHO classification [31].

Generally, angiomyxomas are categorised either as superficial or AAM. Superficial angiomyxoma, also known as cutaneous myxoma, looks like a polypoid cutaneous mass arising from the superficial tissues. This lesion is observed predominantly in middle-aged male adults and may occur in the setting of the Carney complex [19].

The pathogenetic basis is still uncertain, but a translocation at chromosome 12 has been detected in the 12q15 region, which causes an aberrant expression of the high-mobility group protein isoform I-C (HMGI-C) involved in DNA transcription [32].

Differential diagnosis includes angiomyoblastoma, myxoid neurofibroma, myxoma, spindle cell lipoma, myxoid liposarcoma, leiomyosarcoma, and botryoid rhabdo-myosarcoma [33].

Evidence suggests that AAM affects almost exclusively the genital, perineal, and pelvic regions in women of reproductive age. The epidemiological and immunohistochemical characteristics of AAM seem to be related to a hormonal correlation [34]. Immunohistochemical investigation shows positivity for estrogen receptor (ER), progesterone receptor (PR), desmin, smooth muscle actin, and vimentin; some tumours are also CD34-positive [35]. Furthermore, AAM seems to grow in size in pregnant women due to the positivity of progesterone receptors [36].

Hormonal correlation suggests gonadotrophin-releasing hormone (GnRH) agonist or antihormonal therapy (tamoxifen) as emerging therapies, either as an adjuvant approach for residual mass or before surgery to minimize tumour size, and may increase the chance of complete excision [37]. However, adverse effects of long-term use of the GnRH agonist (e.g., menopausal symptoms and bone loss) and tumour regrowth after drug discontinuation do not confirm it as a definitive choice of treatment [27].

An alternative hormone therapy in postmenopausal women is the oral administration of aromatase inhibitors [23].

Finally, angiographic embolization or even chemoembolization has been described; however, alternative blood vessels could provide vascularization of the neoplasm [31].

Wide surgical excision with tumour-free margins remains the preferred approach, but it requires long-term follow-up because of the high frequency of recurrence of AAM. The recurrence rate of AAM after surgery is between 36 and 72%, and it can occur at the same site as the initial resection [38].

The radiological study of neoplasm is significant to determine the actual extension of the tumour; it is also useful for surgical planning and recurrence monitoring. Indeed, it is usually slow-growing and locally infiltrative, extending insidiously into adjacent soft tissues but rarely invading pelvic organs. The infiltrative behaviour and the absence of a well-defined capsule make complete removal difficult and contribute to the high rate of tumour recurrence [8].

MRI with contrast enhancement by gadolinium is the best radiological option for diagnosis, especially on T2-weighted images, which show the lesion as hyperintense relative to muscle [39].

The prognosis is excellent, and metastasis is rarely described in the literature, thanks to the low mitotic index [40].

Despite the potential effect of medical therapies [41], in our patient, we opted for immediate surgery considering her discomfort and blood loss. A subsequent radicalization surgery was necessary because of the positivity of tumour tissue in the surgical resection margins, which confirmed the local infiltrative trend of AAM. Considering the absence of consensus on the role of radical surgery for this type of tumour, we explained to the patient the risks and benefits of a subsequent radicalization surgery vs. medical management in case of positivity for tumour tissue in the surgical resection margins, and the patient accepted the surgical option.

The strength of our work is the description of a rare but insidious tumour that affects women of reproductive age. The knowledge of AAM’s behaviour may guide the surgeon to carry out scrupulous radicalization to prevent local recurrences. Moreover, we realized an extensive review of the literature of the last ten years to analyse which is the best management strategy for this condition.

The main limitation of this study is the heterogeneity of management of this condition regarding medical therapy vs. long-term observation after surgery. Further research on this topic is needed.

## 5. Conclusions

Aggressive angiomyxoma is a very rare neoplasm, and its incidence is predominant among women of reproductive age. The infiltrative behaviour and the high risk of local recurrence make it insidious. Wide surgical excision is the gold standard for treatment, succeeded by either clinical or radiological (ultrasound or MRI) follow-up. However, hormonal therapy as a neo-adjuvant or adjuvant strategy may play a role in management.

## Figures and Tables

**Table 1:** Review of literature of the last 10 years.

Author, Year	Clinical Aspects	History	Symptoms	Site	Treatment	Follow-up
Aguilar-Frasco et al., 2018 [6]	39 yrs; bulky, soft, and mobile bilobulated tumour with dimensions of 28.1 × 10.4 cm	R After 1 year	Tumour	Right labium majus	Surgical excision	GnRH-analogue therapy > 3 months
Brzezinska et al., 2018 [7]	47 yrs; bulky, soft, and mobile tumour with dimensions of 4.3 × 3.6 cm	P	Tumour	Right labium majus	Surgical excision	Observation
Zamani et al., 2021 [8]	28 yrs; bulky, soft pedunculated tumour with dimensions of 20 × 15 × 10 cm.	R After 2 years	Tumour	Vulva and pubis	Surgical excision	GnRH-analogue therapy for 3 months
Xie et al., 2019 [9]	22 yrs; bulky, soft, pedunculated, and ulcerated tumour with dimensions of 30 cm × 20 cm	P	Tumour, pruritus	Left vulva	Surgical excision	Observation
Muskan et al., 2022 [10]	19 yrs; bulky, soft tumour with dimensions of 10 cm × 10 cm	P	Tumour	Left vulva	Surgical excision	Observation
Goyal et al., 2022 [11]	40 yrs; bulky, soft pedunculated and ulcerated tumour 8 × 8 cm	P	Tumour, serosanguinous discharge from the ulceration	Left labium majus	Surgical excision	GnRH-analogue therapy for 3 months
Zhao et al., 2018 [12]	(1) 40 yrs; bulky, soft tumour (2) 38 yrs; bulky soft tumour with dimensions of 7.2 × 5.6 × 14.6 cm (3) 40 yrs; bulky, soft tumour with dimensions of 17.1 × 10.6 × 8.9 cm (4) 35 yrs; bulky, soft tumour with dimensions of 16.2 × 6.9 × 7.4 cm (5) 38 yrs; bulky, soft tumour (6) 45 yrs; bulky, soft tumour with dimensions of 12.3 × 8.8 × 6.3 cm (7) 34 yrs; bulky, soft tumour measuring 11 cm	(1) P (2) R After 2 years (3) P (4) P (5) R After 3 years (6) P (7) P	(1) Tumour (2) Tumour (3) Tumour (4) Tumour (5) Tumour (6) Tumour (7) Tumour	(1) Left vulva (2) Right vulva (3) Left labium majus (4) Left labium majus (5) Left vulva (6) Left vulva (7) Left vulva	(1) Surgical excision (2) Surgical excision (3) Surgical excision (4) Surgical excision (5) Surgical excision (6) Surgical excision (7) Surgical excision	(1) Observation (2) Observation (3) Observation (4) GnRH-analogue therapy (5) Observation (6) Observation (7) Observation
Fatušić et al., 2015 [13]	57 yrs; bulky, soft ulcerated tumour	P	Tumour	Left vulva	Surgical excision	Observation
Ribeiro et al., 2015 [14]	42 yrs; bulky, soft tumour	P	Tumour, difficulty in urinating	Vulva	Surgical excision	Observation
Tariq et al., 2014 [15]	40 yrs; bulky, soft tumour	P	Tumour, pain	Lower-left pelvic-perineal and medial gluteal region	Surgical excision	Observation
Narayama et al., 2016 [16]	49 yrs; bulky, soft tumour with dimensions of 15 × 9.5 × 9 cm	P	Tumour	Left vulva	Surgical excision	Observation
Amin et al., 2013 [17]	89 yrs; bulky, pedunculated tumour with dimensions of 12 × 7 × 6 cm	P	Tumour	Left labium majus	Surgical excision	Observation
R. Elkattah et al., 2013 [18]	38 yrs; bulky, soft tumour with dimensions of 12 × 6 × 4 cm	P	Tumour, dyspareunia	Left labium majus	Surgical excision	Observation
Zizi-Sermpetzoglou et al., 2015 [19]	47 yrs; bulky, soft tumour with dimensions of 25 × 20 × 6 cm	P	Tumour	Right labium majus	Surgical excision	Reoperation 18 months later for recurrence
Sengupta et al., 2014 [20]	34 yrs; bulky, soft tumour with dimensions of 5 × 4 cm	R After 2 years	Tumour	Left labium majus	Surgical excision	Observation
Narang et al., 2014 [21]	40 yrs; bulky, soft tumour with dimensions of 15 × 12 cm	R After 16 months	Tumour	Perineum	Surgical excision	Observation
Das et al., 2016 [22]	40 yrs; bulky, soft tumour with dimensions of 18 × 10 cm	P	Tumour	Right labium majus	Surgical excision	Observation
Schwartz et al., 2014 [23]	32 yrs; bulky, soft tumour with dimensions of 4 × 2.5 cm	R A lot of recurrence during 16 years	Tumour	Left labium majus	Surgical excision	GnRH-analogue therapy for 5 years
Huang et al., 2013 [24]	44 yrs; bulky, soft tumour with dimensions of 12 × 9 × 5 cm	P	Tumour	Left vulva	Surgical excision	Observation
Kiran et al., 2013 [25]	57 yrs; bulky, soft tumour with dimensions of 25 × 30 cm	R After 20 years	Tumour	Left vulva	Surgical excision	Observation
Lee et al., 2014 [26]	35 yrs; bulky, soft tumour with dimensions of 10 × 7 cm	P	Tumour	Right labium majus	Surgical excision	Observation
Choi et al., 2015 [27]	(1) 49 yrs; bulky, soft tumour with dimensions of 19 × 19 cm (2) 31 yrs; mixed echogenic mass of the pelvic cavity with dimensions of 18 × 15 × 8 cm (3) 36 yrs; bulky, soft tumour with dimensions of 15 × 10 × 6 cm	(1) P (2) P (3) P	(1) Tumour (2) Abdominal distension and lower abdominal swelling. (3) Tumour	(1) Left labium majus (2) Pelvis (3) Left buttock	(1) Surgical excision (2) Surgical excision (3) Surgical excision	(1) Observation (2) Observation (3) Observation
Lourenço et al., 2013 [28]	47 yrs; bulky, soft tumour with dimensions of 9 × 7 cm	P	Tumour	Left labium majus	Surgial excision	Observation
Foust-Wright et al., 2012 [29]	19 yrs; soft tumour measuring 3.4 cm	P	Tumour, dysuria	Periurethral mass extending beyond the hymen	Surgical excision	Observation
Ota et al., 2013 [30]	28 yrs; bulky, soft tumour with dimensions of 4.8 × 4 cm	P	Tumour, dysuria	Left labium majus	Surgical excision	Observation

## Data Availability

Data are contained within the article.
